# Factor structure of the University Personality Inventory in Japanese medical students

**DOI:** 10.1186/s40359-020-00469-3

**Published:** 2020-10-01

**Authors:** Norio Sugawara, Norio Yasui-Furukori, Masayuki Sayama, Kazutaka Shimoda

**Affiliations:** 1 Health Services Center for Students and Staff, Dokkyo Medical University, 880 Kitakobayashi, Mibu, Tochigi, 321-0293 Japan; 2 Department of Psychiatry, Dokkyo Medical University School of Medicine, 880 Kitakobayashi, Mibu, Tochigi, 321-0293 Japan

**Keywords:** University personality inventory, Medical students, Exploratory factor analysis, Confirmatory factor analysis

## Abstract

**Background:**

The age of onset for most mental disorders is typically young adulthood, and the university setting is an important one for addressing mental health. The University Personality Inventory (UPI), which was developed to detect mental health problems in university students, is widely used for screening in Japan. However, there have been limited reports on the factor structure of the UPI based on a statistical test for binary indicators. The objective of this study was to assess the factor structure of the UPI in Japanese medical students.

**Methods:**

This study examined the factor structure of the UPI in a sample of 1185 Japanese medical students at the time of university admission. The students were divided into subgroup A (*n* = 589) and subgroup B (*n* = 596) according to their year of university admission. Based on tetrachoric correlation coefficients, exploratory factor analysis (EFA) with promax rotation was applied to explore the dimensions of the inventory in subgroup A. Confirmatory factor analysis (CFA) was then conducted to verify the dimensions in subgroup B.

**Results:**

The EFA with categorical variables yielded four factors in subgroup A. These factors, accounting for 48.9% of the variance, were labeled “Depression and Irritability”, “Anxiety and Persecutory Belief”, “Physical Symptoms”, and “Dependence”. The new four-factor structure showed good fit, and traditional factor structures previously reported were replicated via CFA. The internal consistency reliability was good for the overall UPI scale (alpha = 0.97) and for its four new factors (alpha = 0.83–0.91).

**Conclusions:**

The UPI is a valid and reliable measure that can be used to assess symptoms across four dimensions of mental health in university settings. These findings offer a starting point for the detection of individuals with mental health problems.

## Background

The age of onset for most mental disorders is typically young adulthood [1]. In Japan, more than half of young adults receive postsecondary education [2], and universities are an important setting for addressing mental health. Approximately half of university students are living away from home for the first time and face academic pressure as they study for a degree [3]. Surveys of student life indicate that in addition to academic pressure, university students encounter a multitude of stressors related to financial strains, career choice, and friendship [3]. Compared to the general population, university students might have poorer health-related quality of life [4], and their mental health is more of a problem than their physical health [5]. Although mental illness is prevalent in university students [6, 7], a nonnegligible number of students are reluctant to use mental health services [8] and do not receive adequate treatment [9]. Previous studies have shown that mental health in university students could affect not only their grades but also their intention to drop out [4, 10]. Given the relationship between academic outcomes and mental health, screening for and treating mental health problems have been proposed to promote mental health in university settings.

The University Personality Inventory (UPI), which was developed to assess the mental health status of university students in 1966, has been widely adopted in universities in Japan [11]. The UPI is a 60-item self-report questionnaire that uses a binary scale. The existing literature supports the reliability and convergent validity of this scale [12–14]. Students with a UPI total sum score above 20 or those who respond “yes” to item 25 (“Have an idea of wanting to die”) are identified and guided to arrange personal interviews with mental health professionals [11]. However, mental health problems are heterogeneous and are expressed as a combination of emotional, physical, and social complaints [15]. Traditionally, the UPI has been regarded as a multidimensional instrument for assessing symptoms across four or five domains: physical symptoms, depression, anxiety, neuroticism, persecutory beliefs, and obsessive-compulsive symptoms [11]. However, it has been half a century since the UPI was developed in Japan. Differences in social norms and the degree of westernization could cause psychological distress specific to modern life [16] and affect the factor structure of an instrument that assesses the mental health status of Japanese university students. Furthermore, there have been limited reports on the factor structure of the UPI based on a statistical test for binary indicators [11, 17]. Although a recent report from China found a new five-factor structure consisting of physical symptoms, cognitive symptoms, emotional vulnerability, social avoidance, and interpersonal sensitivity [17], social differences make it difficult to extrapolate the mental health status of Japanese students from the results of a Chinese sample. In addition, the 60-item measurement tool might be lengthy and onerous despite the UPI scale’s established reliability. Brief measurement devices can alleviate respondent burden and lower refusal rates in surveys. It is thus necessary to assess the factor structure of the UPI and suggest the brief version for use among Japanese university students.

This study focuses on medical students, who experience a stressful environment characterized by an increasing study load due to the demanding medical curriculum [18]. In Japan, increasing numbers of students are dropping out of medical school, which is an important issue [19]. A systematic review concerning mental health among medical students indicated that their levels of psychological distress are consistently higher than in the general population [20]. The objective of this study was to assess the factor structure of the UPI in first-year medical students in Japan. To our knowledge, this study is the first to examine the factor structure of the UPI based on a statistical test for binary indicators of the scale.

## Methods

### Participants

This study was conducted between April 2010 and April 2019. The surveys were distributed to 1188 medical students in April of their first year at Dokkyo Medical University School of Medicine. Of the 1188 distributed surveys, 1185 questionnaires (749 males and 436 females) were completed. The demographic data (age and sex) were obtained from a self-report questionnaire. The 1185 students were divided into two subgroups according to their year of university admission. Subgroup A (*n* = 589; 372 males and 217 females) consisted of students who entered the university in an even-numbered year, and subgroup B (*n* = 596; 377 males and 219 females) consisted of students who entered the university in an odd-numbered year.

### Measures

The UPI is a 60-item self-report measure assessing whether an individual usually experienced the described symptom during the past year [11]. For each item, a score of 1 was given for “Yes”, and 0 was given for “No”. After excluding the lie scales (items 5, 20, 35, and 50), we analyzed the 56 items describing psychosomatic problems. Traditionally, the 56-item UPI is regarded as a multidimensional instrument with as many as four or five factors [11]. The higher the score, the poorer the mental and/or physical condition.

### Statistical analysis

Based on tetrachoric correlation coefficients, an EFA for binary indicators was conducted with promax rotation to analyze the underlying structure of the UPI in subgroup A. Because previous studies showed interfactor correlations in the factor structure of the UPI, we used promax rotation, which allows the factors to be correlated. We determined the number of factors to retain based on eigenvalues, the scree test, and the interpretability of the factors; four factors were retained. Furthermore, confirmatory factor analysis (CFA) was conducted to verify the dimensions in subgroup B. Five practical fit indices were used to evaluate the model fit: the goodness of fit index (GFI), the adjusted goodness of fit index (AGFI), the root mean square error of approximation (RMSEA), and the comparative fit index (CFI). A GFI, AGFI and CFI close to 1 indicate a good fit. An RMSEA < 0.05 indicates good fit. The data analysis was performed using R for Windows, Version 3.6.3 (The R Foundation for Statistical Computing, Vienna, Austria) [21].

## Results

The mean (± standard deviation) age of the study participants was 19.6 ± 1.7 years (subgroup A: 19.6 ± 1.7; subgroup B: 19.5 ± 1.6). The overall reliability of the scale was good (alpha = 0.97). Corrected item-total correlations for individual items ranged from 0.37 (item 31, “Distressed by blushing”) to 0.80 (item 13, “Pessimistic”). The EFA with categorical variables yielded four factors in subgroup A. Factors 1 through Factor 4 were tentatively labeled “Depression and Irritability”, “Anxiety and Persecutory Belief”, “Physical Symptoms”, and “Dependence”. These factors accounted for 48.9% of the variance. Table 1 presents the rotated factor loadings for the new four-factor model. Twenty-six items had low loadings: 3, 4, 7, 9, 11, 13, 14, 15, 16, 22, 27, 28, 32, 34, 36, 37, 40, 42, 44, 47, 49, 51, 53, 54, 59 and 60.

**Table 1:** Factor loadings in the exploratory factor analysis of the university personality inventory

Item		Factor 1	Factor 2	Factor 3	Factor 4
1	Poor appetite	0.016	− 0.056	**0.664**	0.106
2	Feel sick, stomachache	0.003	0.013	**0.761**	0.019
3	Easily have diarrhea or constipation	−0.145	0.183	0.388	0.139
4	Care about palpitation and pulse	−0.129	0.393	0.404	0.018
6	Full of dissatisfaction and complaints	**0.693**	0.145	−0.011	0.013
7	High expectation from parents	0.483	0.038	0.039	−0.126
8	My past and family is misfortune	**0.766**	0.050	−0.183	−0.154
9	Over-worry about my future	0.177	0.214	−0.059	0.406
10	Do not like meeting others	**0.584**	−0.006	0.200	0.083
11	Feel that I am not myself	0.285	0.238	0.068	0.349
12	Lack of enthusiasm and positivity	0.470	−0.271	**0.538**	0.184
13	Pessimistic	0.358	0.152	0.185	0.282
14	Distracted	0.164	0.119	0.224	0.405
15	Over-uneven in emotion	0.430	0.264	0.042	0.110
16	Frequent insomnia	0.245	0.072	0.230	0.021
17	Headache	0.098	−0.018	**0.691**	−0.076
18	Ache in neck and shoulder	−0.043	− 0.001	**0.632**	− 0.096
19	Chest pain or feel oppressed	0.040	0.132	**0.545**	0.050
21	Intolerance	0.048	0.409	−0.255	**0.576**
22	Inclined to worry	0.234	0.291	0.126	0.235
23	Restless	**0.522**	0.327	0.033	−0.061
24	Irritable	**0.657**	0.329	−0.074	−0.112
25	Have idea of wanting to die	**0.685**	0.086	−0.045	0.051
26	No interest in anything	**0.523**	−0.227	0.178	0.452
27	Declining memory	0.235	0.141	0.217	0.172
28	Lack of patience	0.339	−0.160	0.284	0.354
29	Lack of judgment	−0.151	−0.008	0.090	**0.833**
30	Too dependent on others	0.080	0.143	−0.097	**0.567**
31	Distressed by blushing	−0.156	**0.516**	0.069	−0.029
32	Stuttering, faltering voice	0.128	0.309	0.270	0.083
33	Feel hot and cold	−0.357	0.377	**0.660**	0.004
34	Concern about urination or sexual organs	0.352	0.404	0.107	−0.294
36	Uneasy without reason	0.194	0.225	0.028	0.437
37	Feel uneasy when alone	−0.046	0.336	0.026	0.250
38	Lack of confidence	0.007	0.029	0.155	**0.753**
39	Irresolute about anything	−0.119	0.114	−0.063	**0.837**
40	Easily feel misunderstood	0.450	0.449	−0.079	−0.104
41	Lack faith in others	**0.560**	0.036	0.063	0.141
42	Over-suspicious	0.047	0.411	0.007	0.265
43	Unwilling to associate with others	**0.644**	−0.162	0.038	0.185
44	Feel self-abased	0.222	0.280	−0.001	0.384
45	Catastrophizing	0.005	**0.521**	0.032	0.207
46	Physically exhausted	0.172	−0.191	**0.656**	0.238
47	In cold sweat when I hurry	−0.225	0.460	0.157	0.114
48	Dizzy when I stand up	0.045	0.031	**0.745**	−0.215
49	Have ever lost consciousness, cramp	0.155	0.309	0.190	−0.283
51	Over-rigid	0.167	0.365	−0.039	0.060
52	Cannot give up repeating things	−0.055	**0.506**	−0.154	0.254
53	Susceptible to dirtiness	0.052	0.417	0.154	−0.034
54	Cannot get rid of meaningless idea	0.272	0.382	−0.085	0.286
55	Sense weird smell from myself	0.151	**0.551**	−0.059	0.029
56	Suspect others say something bad about me	0.434	**0.538**	0.158	−0.367
57	Wary of others	0.214	**0.600**	−0.202	0.325
58	Care about others’ gaze	0.087	**0.638**	−0.101	0.268
59	Feel others despise me	0.341	0.217	0.201	0.145
60	Sensitive emotions	0.432	0.401	−0.115	0.104
	Interfactor correlations				
	Factor 1	1.000			
	Factor 2	0.567	1.000		
	Factor 3	0.567	0.567	1.000	
	Factor 4	0.616	0.533	0.464	1.000

The loadings of 0.50 or above are boldfaced

After excluding the 26 items with low loadings, a CFA was conducted on the new four-factor model with the remaining 30 items in subgroup B. The factor loadings for the new four-factor model are shown in Table 2. The alpha coefficients for the four new factors were 0.91 for “Depression and Irritability”, 0.83 for “Anxiety and Persecutory Belief”, 0.89 for “Physical Symptoms” and 0.90 for “Dependence”. Intercorrelations between the four factors in the new four-factor model ranged from 0.55 to 0.77. For the traditional four-factor model, CFA was conducted on the 56 items in subgroup B. The factor loadings for the traditional four-factor model are shown in Table 3. The alpha coefficients for the traditional four factors were 0.89 for “Physical Symptoms”, 0.94 for “Depression”, 0.90 for “Anxiety” and 0.89 for “Neuroticism and Persecutory Beliefs”. Intercorrelations between the four factors in the traditional four-factor model ranged from 0.69 to 0.96. For the traditional five-factor model, CFA was conducted on the 56 items in subgroup B. The factor loadings for the traditional five-factor model are shown in Table 4. The alpha coefficients for the traditional five factors were 0.89 for “Physical Symptoms”, 0.94 for “Depression”, 0.90 for “Anxiety”, 0.78 for “Obsessive-compulsive” and 0.87 for “Persecutory Beliefs”. Intercorrelations between the five factors in the traditional five-factor model ranged from 0.60 to 0.96. Table 5 shows the fit indices for the CFA models.

**Table 2:** Factor loadings for new four-factor model in the confirmatory factor analysis of the university personality inventory

Item		New four-factor model			
		Factor 1	Factor 2	Factor 3	Factor 4
6	Full of dissatisfaction and complaints	0.585			
8	My past and family is misfortune	0.353			
10	Do not like meeting others	0.617			
23	Restless	0.581			
24	Irritable	0.565			
25	Have idea of wanting to die	0.523			
26	No interest in anything	0.552			
41	Lack faith in others	0.613			
43	Unwilling to associate with others	0.553			
31	Distressed by blushing		0.356		
45	Catastrophizing		0.539		
52	Cannot give up repeating things		0.458		
55	Sense weird smell from myself		0.398		
56	Suspect others say something bad about me		0.462		
57	Wary of others		0.703		
58	Care about others’ gaze		0.644		
1	Poor appetite			0.527	
2	Feel sick, stomachache			0.541	
12	Lack of enthusiasm and positivity			0.724	
17	Headache			0.519	
18	Ache in neck and shoulder			0.424	
19	Chest pain or feel oppressed			0.481	
33	Feel hot and cold			0.457	
46	Physically exhausted			0.728	
48	Dizzy when I stand up			0.408	
21	Intolerance				0.584
29	Lack of judgment				0.629
30	Too dependent on others				0.585
38	Lack of confidence				0.774
39	Irresolute about anything				0.707
	Interfactor correlations				
	Factor 1	1.000			
	Factor 2	0.683	1.000		
	Factor 3	0.692	0.624	1.000	
	Factor 4	0.618	0.769	0.554	1.000

The factor 1 was labelled the “Depression and Ittitability” factor

The factor 2 was labelled the “Anxiety and Persecutory belief” factor

The factor 3 was labelled the “Physical symptoms” factor

The factor 4 was labelled the “Dependence” factor

**Table 3:** Factor loadings for traditional four-factor model in the confirmatory factor analysis of the university personality inventory

Item		Traditional four-factor model			
		Factor 1	Factor 2	Factor 3	Factor 4
1	Poor appetite	0.544			
2	Feel sick, stomachache	0.591			
3	Easily have diarrhea or constipation	0.412			
4	Care about palpitation and pulse	0.395			
16	Frequent insomnia	0.400			
17	Headache	0.506			
18	Ache in neck and shoulder	0.356			
19	Chest pain or feel oppressed	0.473			
31	Distressed by blushing	0.270			
32	Stuttering, faltering voice	0.465			
33	Feel hot and cold	0.490			
34	Concern about urination or sexual organs	0.354			
46	Physically exhausted	0.662			
47	In cold sweat when I hurry	0.358			
48	Dizzy when I stand up	0.432			
49	Have ever lost consciousness, cramp	0.102			
6	Full of dissatisfaction and complaints		0.573		
7	High expectation from parents		0.253		
8	My past and family is misfortune		0.217		
9	Over-worry about my future		0.502		
10	Do not like meeting others		0.519		
11	Feel that I am not myself		0.490		
12	Lack of enthusiasm and positivity		0.621		
13	Pessimistic		0.674		
14	Distracted		0.622		
15	Over-uneven in emotion		0.563		
21	Intolerance		0.521		
22	Inclined to worry		0.579		
23	Restless		0.568		
24	Irritable		0.506		
25	Have idea of wanting to die		0.408		
26	No interest in anything		0.529		
27	Declining memory		0.502		
28	Lack of patience		0.546		
29	Lack of judgment		0.505		
30	Too dependent on others		0.469		
36	Uneasy without reason			0.564	
37	Feel uneasy when alone			0.317	
38	Lack of confidence			0.638	
39	Irresolute about anything			0.525	
40	Easily feel misunderstood			0.466	
41	Lack faith in others			0.496	
42	Over-suspicious			0.501	
43	Unwilling to associate with others			0.419	
44	Feel self-abased			0.627	
45	Catastrophizing			0.527	
51	Over-rigid				0.396
52	Cannot give up repeating things				0.394
53	Susceptible to dirtiness				0.351
54	Cannot get rid of meaningless idea				0.623
55	Sense weird smell from myself				0.429
56	Suspect others say something bad about me				0.416
57	Wary of others				0.697
58	Care about others’ gaze				0.639
59	Feel others despise me				0.475
60	Sensitive emotions				0.596
	Interfactor correlations				
	Factor 1	1.000			
	Factor 2	0.753	1.000		
	Factor 3	0.712	0.959	1.000	
	Factor 4	0.690	0.897	0.939	1.000

The factor 1 was labelled the “Physical symptoms” factor

The factor 2 was labelled the “Depression” factor

The factor 3 was labelled the “Anxiety” factor

The factor 4 was labelled the “Neuroticism and persecutory beliefs” factor

**Table 4:** Factor loadings for traditional five-factor model in the confirmatory factor analysis of the university personality inventory

Item		Traditional five-factor model				
		Factor 1	Factor 2	Factor 3	Factor 4	Factor 5
1	Poor appetite	0.544				
2	Feel sick, stomachache	0.591				
3	Easily have diarrhea or constipation	0.412				
4	Care about palpitation and pulse	0.395				
16	Frequent insomnia	0.400				
17	Headache	0.506				
18	Ache in neck and shoulder	0.356				
19	Chest pain or feel oppressed	0.473				
31	Distressed by blushing	0.270				
32	Stuttering, faltering voice	0.465				
33	Feel hot and cold	0.490				
34	Concern about urination or sexual organs	0.355				
46	Physically exhausted	0.662				
47	In cold sweat when I hurry	0.358				
48	Dizzy when I stand up	0.432				
49	Have ever lost consciousness, cramp	0.102				
6	Full of dissatisfaction and complaints		0.573			
7	High expectation from parents		0.253			
8	My past and family is misfortune		0.217			
9	Over-worry about my future		0.502			
10	Do not like meeting others		0.519			
11	Feel that I am not myself		0.490			
12	Lack of enthusiasm and positivity		0.621			
13	Pessimistic		0.674			
14	Distracted		0.622			
15	Over-uneven in emotion		0.563			
21	Intolerance		0.521			
22	Inclined to worry		0.579			
23	Restless		0.568			
24	Irritable		0.506			
25	Have idea of wanting to die		0.408			
26	No interest in anything		0.529			
27	Declining memory		0.502			
28	Lack of patience		0.546			
29	Lack of judgment		0.505			
30	Too dependent on others		0.469			
36	Uneasy without reason			0.564		
37	Feel uneasy when alone			0.317		
38	Lack of confidence			0.638		
39	Irresolute about anything			0.525		
40	Easily feel misunderstood			0.466		
41	Lack faith in others			0.496		
42	Over-suspicious			0.501		
43	Unwilling to associate with others			0.419		
44	Feel self-abased			0.627		
45	Catastrophizing			0.527		
51	Over-rigid				0.452	
52	Cannot give up repeating things				0.447	
53	Susceptible to dirtiness				0.400	
54	Cannot get rid of meaningless idea				0.710	
55	Sense weird smell from myself					0.436
56	Suspect others say something bad about me					0.423
57	Wary of others					0.709
58	Care about others’ gaze					0.650
59	Feel others despise me					0.483
60	Sensitive emotions					0.607
	Interfactor correlations					
	Factor 1	1.000				
	Factor 2	0.753	1.000			
	Factor 3	0.712	0.959	1.000		
	Factor 4	0.601	0.786	0.824	1.000	
	Factor 5	0.681	0.883	0.923	0.796	1.000

The factor 1 was labelled the “Physical symptoms” factor

The factor 2 was labelled the “Depression” factor

The factor 3 was labelled the “Anxiety” factor

The factor 4 was labelled the “Obsessive-compulsive” factor

The factor 5 was labelled the “Persecutory beliefs” factor

**Table 5:** Fit indices for confirmatory factor models

	GFI	AGFI	RMSEA	CFI
One factor model	0.999	0.999	0.034	0.976
New four-factor model	1.000	1.000	0.034	0.980
Traditional four-factor model	0.997	0.996	0.027	0.985
Traditional five-factor model	0.997	0.996	0.027	0.985

*GFI* goodness of fit index, *AGFI* adjusted goodness of fit index, *RMSEA* root mean square error of approximatin, *CFI* comparative fit index

## Discussion

The aim of the present study was to examine the factor structure of the UPI among Japanese medical students. In our sample, the good internal consistency of the overall UPI (alpha = 0.97) indicated that a total score of this scale can be used as a global indicator of psychological distress. In subgroup A, we demonstrated that the UPI consists of four factors via EFA with categorical variables. These factors, accounting for 48.9% of the variance, were labeled “Depression and Irritability”, “Anxiety and Persecutory Belief”, “Physical Symptoms”, and “Dependence”. Furthermore, the new four-factor structure showed good fit, and traditional factor structures previously reported were replicated by CFA in subgroup B.

With regard to the EFA, a previous study based on a statistical test for binary indicators found a new five-factor structure in Chinese students [17]. The factors “Physical Symptoms” and “Cognitive Symptoms” in that study are comparable to the factors that we labeled “Physical Symptoms” and “Dependence”, respectively. However, the UPI items belonging to the “Depression and Irritability” and “Anxiety and Persecutory Belief” factors in our new four-factor model constitute different factors in the Chinese study. The different response patterns between Japanese and Chinese individuals may be due to ethnicity or the social environment. In addition, the premorbid personality of so-called “*Shin-gata utsu-byo*” [new-type depression (NTD)] might affect our results. In Japan, depression characterized by a premorbid personality different from the traditional melancholic temperament has been reported among young adults since approximately 2000 [22]. Initially, Tarumi called this novel depression “dysthymic-type” and advocated that the premorbid personality and symptomatologic features of NTD include avoidant narcissistic personality, extrapunitive feelings, and stress related to social rules and expectations [22, 23]. The “Depression and Irritability” and “Anxiety and Persecutory Belief” factors might be premorbid features of NTD reflecting extrapunitive feelings and stress related to social rules and expectations. Furthermore, avoidant narcissistic personality might also contribute to the “Dependence” factor. In Japanese students, subclinical symptoms of depression and anxiety could be accompanied by anger, avoidance, or dependence.

In psychological evaluation, somatic symptoms are generally considered manifestations of underlying psychological distress, such as anxiety or depression [11, 15]. Previous studies found via EFA that items of emotional and physical symptoms merged and constituted new factors in Asian or Asian-American populations [15, 24–26]. However, exploratory analysis of the UPI did not show such merging of emotional and physical symptoms in either Japanese or Chinese students [17]. Discrepant responses between the UPI and other psychological measures might be explained by differences in participants’ age. Because most studies employing the UPI focus on university students, participants in such studies are typically in their late teens or early 20s [11, 17]. Another explanation is that differences in items or expressed statements could affect the results.

The good fit of the CFA models of the UPI (Table 5) supports the use of all the models suggested in our study as indicators for psychological distress. However, both four-factor and five-factor traditional models of the UPI showed high interfactor correlations (> 0.95) between “Depression” and “Anxiety” in. In the same models, anxiety was also highly correlated with “Neuroticism and Persecutory Beliefs” (0.94) or “Persecutory Beliefs” (0.92). Although the structures of the abovementioned factors might have been distinct in Japanese students in the 1960s, they are not in students in the twenty-first century.

## Limitations

The current study has some limitations. First, subject recruitment was restricted to medical students. Medical students are known to be at high risk for depression and suicidal ideation [27, 28]. In addition, students’ university major could affect the response pattern on the UPI [11]. We cannot generalize our findings to all university students. Second, due to the lack of data on clinical diagnoses or other psychological measures, we could not confirm the criterion validity of the UPI. These limitations should be addressed in future studies. Third, this research was conducted over a long 9-year period, and some underlying psychosocial factors may change over time.

## Conclusion

This study found a four-factor structure of the UPI by EFA in Japanese medical students. In Japan, this is the first study on the factor structure of the UPI based on a statistical test for binary indicators. Furthermore, CFA confirmed that the new four-factor structure as well as traditional factor structures previously reported showed good fit. The good internal consistency of the overall UPI (alpha = 0.97) indicated that a total score of this scale can be used as a global indicator of psychological distress. The UPI is a valid and reliable measure that can be used to assess symptoms in multiple dimensions of mental health in university settings. The new four-factor model of the UPI consisting of 30 items is feasible and adequate psychological measure for modern university students. These findings offer a starting point for the detection of individuals with mental health problems. Future studies with a longitudinal design are needed to investigate the predictive validity of the UPI for mental or academic outcomes in university students.

## Data Availability

All data used and/or analyzed during this study are not publicly available to maintain the anonymity of the individuals concerned. The dataset supporting the conclusions is available from the corresponding author on reasonable request.

## Abbreviations

AGFI: Adjusted Goodness of Fit Index
CFA: Confirmatory Factor Analysis
CFI: Comparative Fit Index
EFA: Exploratory Factor Analysis
GFI: Goodness of Fit Index
NTD: New-Type Depression
RMSEA: Root Mean Square Error of Approximation
UPI: University Personality Inventory
